# Histopathological growth patterns of colorectal liver metastasis exhibit little heterogeneity and can be determined with a high diagnostic accuracy

**DOI:** 10.1007/s10585-019-09975-0

**Published:** 2019-05-27

**Authors:** D. J. Höppener, P. M. H. Nierop, E. Herpel, N. N. Rahbari, M. Doukas, P. B. Vermeulen, D. J. Grünhagen, C. Verhoef

**Affiliations:** 1 Department of Surgical Oncology and Gastrointestinal Surgery, Erasmus MC Cancer Institute, P.O. Box 2040, 3000 CA Rotterdam, The Netherlands; 2 Institute of Pathology, University Hospital Heidelberg, Heidelberg, Germany; 3 Tissue Bank of the National Center for Tumor Diseases (NCT), Heidelberg, Germany; 4 Department of Surgery, Mannheim University Medical Centre, University of Heidelberg, Mannheim, Germany; 5 Department of Pathology, Erasmus MC, Rotterdam, The Netherlands; 6 Translational Cancer Research Unit (GZA Hospitals, University of Antwerp), Antwerp, Belgium

**Keywords:** Colorectal cancer, Colorectal liver metastasis, Histopathological growth pattern, Histological biomarker, External validation, Concordance, Diagnostic accuracy, Interobserver agreement

## Abstract

Colorectal liver metastases (CRLM) exhibit distinct histopathological growth patterns (HGPs) that are indicative of prognosis following surgical treatment. This study aims to assess the reliability and replicability of this histological biomarker. Within and between metastasis HGP concordance was analysed in patients who underwent surgery for CRLM. An independent cohort was used for external validation. Within metastasis concordance was assessed in CRLM with ≥ 2 tissue blocks. Similarly, concordance amongst multiple metastases was determined in patients with ≥ 2 resected CRLM. Diagnostic accuracy [expressed in area under the curve (AUC)] was compared by number of blocks and number of metastases scored. Interobserver agreement (Cohen’s *k*) compared to the gold standard was determined for a pathologist and a PhD candidate without experience in HGP assessment after one and two training sessions. Both the within (95%, n = 825) and the between metastasis (90%, n = 363) HGP concordance was high. These results could be replicated in the external validation cohort with a within and between metastasis concordance of 97% and 94%, respectively. Diagnostic accuracy improved when scoring 2 versus 1 blocks(s) or CRLM (AUC = 95.9 vs. 97.7 [p = 0.039] and AUC = 96.5 vs. 93.3 [p = 0.026], respectively), but not when scoring 3 versus 2 blocks or CRLM (both p > 0.2). After two training sessions the interobserver agreement for both the pathologist and the PhD candidate were excellent (*k *= 0.953 and *k *= 0.951, respectively). The histopathological growth patterns of colorectal liver metastasis exhibit little heterogeneity and can be determined with a high diagnostic accuracy, making them a reliable and replicable histological biomarker.

**Electronic supplementary material:**

The online version of this article (10.1007/s10585-019-09975-0) contains supplementary material, which is available to authorized users.

## Introduction

Colorectal cancer (CRC) is one of the most prevalent solid malignancies in the world with approximately one third of patients developing hepatic metastases [1–5]. Even though surgical treatment is seen as the only potentially curative treatment option, reported 5-year survival rates vary widely (from 20 to 70%) [6–13].

**Fig. 1:**
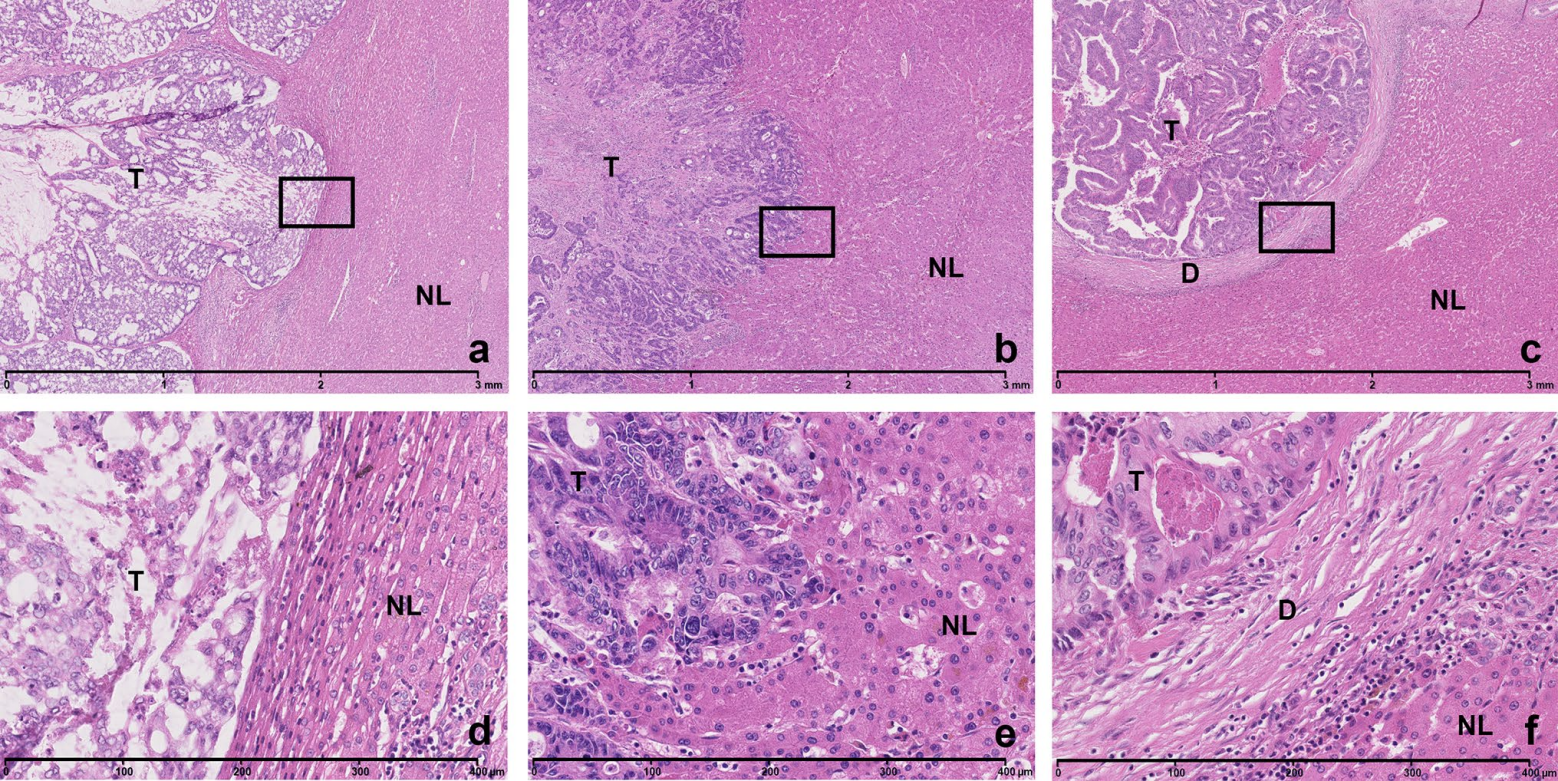
Three distinct types of histopathological growth patterns (HGPs) can be identified on H&E stained tissue blocks. **a**–**c** × 2.5 magnification. **d**–**f** × 20 magnification. **a**, **d** Pushing type HGP. **b**, **e** Replacement type HGP. **c**, **f** Desmoplastic type HGP. *T* tumour, *NL* normal liver, *D* desmoplastic stroma

Recently, a new potential histological biomarker has been described [14, 15]. Colorectal liver metastases (CRLM) grow in three distinct histopathological growth patterns (HGP), the desmoplastic, the replacement and the pushing type, each with unique morphological and biological features (Fig. 1a–f). These distinct features have previously been described in detail [16–18]. In short: HGP assessment is performed by assessing the proportion (expressed as percentage) of each distinct HGP observed at the tumour-liver interface on H&E stained tissue sections [14]. Previous studies suggest that a high relative proportion of the replacement type is prognostic for an impaired overall survival [19–22]. The largest and most recent study analysed a cohort of 732 patients and found that it is the presence rather than the relative proportion of any non-desmoplastic type HGP (i.e. pushing and/or replacement type) that dictates poor prognosis [15]. In terms of clinical relevance, HGPs can therefore be classified into two categories: either pure desmoplastic (dHGP) or any observed non-desmoplastic type HGP (non-dHGP) [15].

While interesting from a biological point of view, this new classification raises methodological concerns. For if classification is based on either 100% dHGP or < 100% dHGP, assessment could be more susceptible to sampling and reading error. In order to validate HGPs as a histological biomarker, knowledge on HGP concordance within a single and amongst multiple metastases within the same patient is essential, especially considering the growing evidence of (non-)genetic intra-tumoural heterogeneity in CRC [23]. Knowledge on diagnostic accuracy and learnability of HGP assessment is also necessitated to determine the reliability and replicability of this histological biomarker. This study therefore analyses within and between metastasis HGP concordance within the same cohort as described by Galjart et al. [15], as well as an external validation cohort [24]. In addition, diagnostic accuracy is determined for scoring a single or multiple formalin-fixed paraffin-embedded (FFPE) tissue blocks per CRLM and for scoring a single or multiple CRLM per patient. Lastly, the learning curve associated with HGP assessment is determined in two observers (pathologist and PhD candidate) without prior experience in HGP assessment.

## Methods

The current study was approved by the medical ethics committee of the Erasmus University Medical Center (MEC-2018-1743). The need for informed consent was waived by the ethics committee due to the retrospective and non-invasive nature of the study. Drafting of the manuscript was performed in accordance with the REMARK guidelines [25].

### Patient selection

The patient selection for the current study was performed in the same cohort as described by Galjart et al. [15]. Patients undergoing resection of CRLM at the Erasmus MC Cancer Institute, the Netherlands, between January 2000 and March 2015 were eligible for inclusion.

### Routine pathological assessment

During macroscopic pathological assessment of the surgical specimens of resected CRLM, representative sections (e.g. tumour, tumour with relation to the surgical margin(s), capsule, background liver, non-tumorous liver in distance) were considered for preparation of FFPE tissue blocks. A 5 µm section per block was cut and stained with Haematoxylin and Eosin (H&E) for pathological interpretation. If needed, deeper levels of the block were cut and stained with H&E.

### Assessment of HGPs

H&E stained slides retrieved from the archive of the Pathology Department of the Erasmus MC were retrospectively reviewed by light microscopy (Fig. 1a–f). Scoring of the HGPs was performed in accordance with international consensus guidelines [14]. For each block subjected to review the relative presence [in percentage (%)] at the tumour-liver interface of the distinct HGP’s (pushing, desmoplastic and replacement type) was estimated. The metastasis HGP was defined as the pooled estimate (average with equal weights per block) of all blocks of a single CRLM. Concordantly, the patient HGP was defined as the pooled estimate (average with equal weights per CRLM) of all resected CRLM within a single patient. Given recent findings [15], block, metastasis and patient HGP were classified as dHGP if only the desmoplastic type was observed (i.e. 100% dHGP), and as non-dHGP if any percentage of pushing and/or replacement type was observed (i.e. < 100% dHGP). Due to this on/off classification, if non-dHGP is observed on a single block, corresponding metastasis and patient HGP is classified as non-dHGP, regardless of the HGP of other blocks within the same metastasis or other CRLM within the same patient.

For the within metastasis analysis, concordance (yes/no) of block HGP to metastasis HGP was recorded for all resected CRLM with ≥ 2 tissue blocks. Within metastasis concordance was defined as the proportion of concordant tissue blocks. Since a lesion represents a three dimensional structure, consecutive slides from a single block (i.e. deeper levels) do not adequately represent its three dimensional nature. As such, consecutive slides from a single block were excluded from the within metastasis analysis. For the between metastasis analysis, concordance (yes/no) of metastasis HGP to patient HGP was determined in all patients with ≥ 2 CRLM resected in a single time-frame (e.g. no recurrent CRLM). Between metastasis concordance was defined as within patient proportion of concordant CRLM. Patient information and data on primary CRC and CRLM were extracted from a prospectively maintained database. Regarding systemic treatment status, patients were considered chemo-naive if they did not receive any form of chemotherapy within the 6 months prior to resection. Multivariable logistic regression analysis was performed for within metastasis discordance (yes/no) with primary tumour characteristics, known clinical risk factors, systemic treatment status and number of blocks scored entered into the model. Significant predictor(s) found for within metastasis discordance were used as stratification factor(s) for between metastasis analysis. Identical models were fitted within each stratum (if applicable) to predict discordance (yes/no) amongst multiple metastases. Mean within metastasis concordance was compared across number of blocks scored. Similarly, mean between metastasis concordance was compared within strata (if applicable) and by number of CRLM resected.

### External validation

External validation of mean within and mean between metastasis concordance was performed by retrospective HGP assessment as described previously. The external validation cohort comprised of chemo-naive patients treated surgically for CRLM at the University Hospital of Heidelberg, Germany, between October 2001 and June 2009 [24]. H&E stained sections of the validation cohort were provided by the tissue bank of the National Center for Tumor Diseases (NCT). As the external validation cohort consisted of chemo-naive patients, comparisons to the original cohort were performed in (tissues from) chemo-naive patients only.

### Diagnostic accuracy

Diagnostic accuracy for scoring a single FFPE block was determined in all CRLM with ≥ 2 blocks. Of these ≥ 2 blocks, one individual block was selected at random. The HGP of this randomly selected block was considered the predictor (i.e. test result), while the metastasis HGP—as determined by HGP assessment of all ≥ 2 blocks of the metastasis in question—was considered the response (i.e. true HGP status). This was done similarly for 2 blocks in all CRLM with ≥ 3 blocks. Identically, the diagnostic accuracy of scoring a single resected CRLM was determined within patients with ≥ 2 CRLM resected etc. The area under the curve [AUC] of the corresponding receiver operating characteristic (ROC) curves were compared for 2 versus 1 block(s) or CRLM scored, and for 3 versus 2 blocks or CRLM scored, respectively.

### Learning curve

A gastro-intestinal pathologist (MD) and a PhD-candidate (DH) without prior pathology experience were recruited for learning curve analysis. Both observers had no prior experience in HGP assessment. The raters received a joint training session by a pathologist with over 10 years of experience in HGP assessment (PV). During this training session, 50 tissue sections were assessed collaboratively. Hereafter, both observers independently scored a test-set of an additional 50 tissue sections. Individual scores of the test-set were reviewed in a joint session with the trainer, followed by a second training session of 50 tissue sections. Subsequently a second test-set of 50 tissue sections was scored independently. After completion scores were again collaboratively reviewed. For both test-sets, interobserver agreement of both observers compared to the gold standard was determined for the dHGP/non-dHGP classification. The scores of the experienced trainer were considered the gold standard.

### Statistical analysis

Dichotomous or categorical data are reported as percentage, parametric continuous data are reported as mean (standard deviation [SD]) and non-parametric continuous data are reported as median (inter-quartile range [IQR]). Mean concordances were compared by an independent samples *T* test or a one-way analysis of variance (ANOVA), depending on the number of strata. AUC values were compared as described by DeLong [26]. Interobserver agreement was determined using Cohen’s kappa. All statistical analyses were performed using R version 3.5.3 (http://www.r-project.org). The R-package ‘pROC’ was used for comparison of AUC values. A p-value < 0.05 was considered statistically significant.

## Results

### Patient characteristics

In total 785 patients underwent resection of one or more CRLM at the Erasmus MC Cancer Institute in the study period and were consequently scored for HGP. In total 1625 CRLM were resected. Of these, 835 CRLM had two or more H&E stained slides available for review (2135 slides in total) and were considered for within metastasis analysis. Of these, 31 slides of ten individual CRLM were identified as consecutively cut from single FFPE blocks, and were hence excluded from within metastasis analysis. Resection of two or more CRLM was performed in 382 patients. Nineteen were excluded for between metastasis analysis due to missing data required to link individual tissue samples to individual CRLM. Within the remaining 363 patients a total of 1118 CRLM were resected. Patient characteristics are reported in Table 1.

**Table 1:** Characteristics of patients included for between metastasis concordance analysis. *CRLM* colorectal liver metastasis, *IQR* interquartile range, *CEA* carcinoembryonic antigen, *CTx* chemotherapy, *RFA* radiofrequency ablation, *MWA* microwave ablation, *(non*-*)dHGP* (non-)desmoplastic type histopathological growth pattern

	n = 363 (%)
Gender	
Female	233 (64)
Male	130 (36)
Age at resection CRLM—[median (IQR)]	63.0 (57.0–70.0)
Primary tumour location	
Right-sided	61 (17)
Left-sided	152 (42)
Rectal	145 (40)
Missing	5 (1)
T-stage	
pT 0–2	70 (19)
pT 3–4	265 (73)
Missing	28 (8)
N-stage	
N0	118 (33)
N+	216 (60)
Missing	29 (8)
Disease-free interval—months [median (IQR)]	0.0 [0.0–9.0]
Diameter of largest CRLM—cm [median (IQR)]	3.1 [2.0–4.8]
Preoperative CEA—µg/L [median (IQR)]	20.0 [5.4–70.1]
Preoperative CTx status	
Chemo-naive	121 (33)
Pre-treated	242 (67)
Two-staged resection	
No	347 (96)
Yes	16 (4)
Use of RFA or MWA	
No	252 (69)
Yes	111 (31)
Number of CRLM resected	
2	175 (48)
3	87 (24)
4	58 (16)
≥ 5	43 (12)
Histopathological growth pattern	
dHGP	72 (20)
Non-dHGP	291 (80)

### Within metastasis concordance

**Fig. 2:**
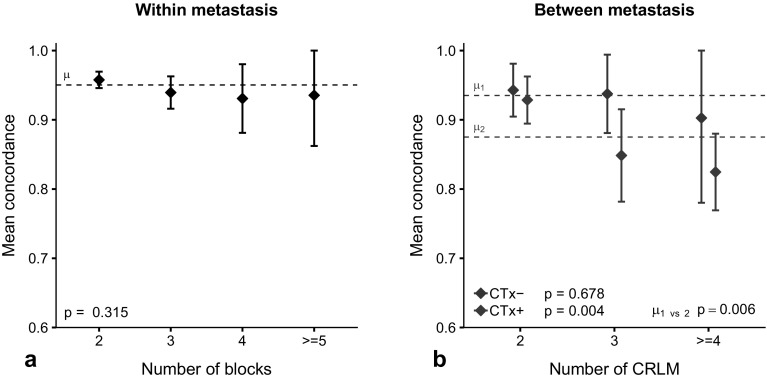
**a** Within metastasis histopathological growth pattern (HGP) concordance within the original cohort stratified by number of blocks scored. Overall mean within metastasis concordance (μ) was 95%. **b** Mean between metastasis HGP concordance within the original cohort stratified by preoperative chemotherapy status and number of colorectal liver metastasis (CRLM) resected. Mean between metastasis concordance in chemo-naive (CTx-) patients was 94% (μ_1_). Mean between metastasis concordance in pre-treated (CTx +) patients was 88% (μ_2_). Error-bars represent the 95% confidence interval of the estimate

Non-dHGP was observed in 72% of reviewed tissue blocks. Results of the multivariable logistic regression model on within metastasis discordance are reported in Table 2. Systemic treatment status proved to be a significant predictor for presence of HGP discordance (yes/no) amongst multiple blocks, with an odds ratio (OR) (95% CI) of 2.107 (1.231; 3.679) and p = 0.007 for pre-treated versus chemo-naive CRLM. Mean within metastasis concordance was 95%. Figure 2a shows the mean within metastasis concordance stratified by number of blocks scored. Mean within metastasis concordance (95% CI) for 2, 3, 4, or ≥ 5 blocks scored was 96% (95; 97), 94% (92; 96), 93% (88; 98) and 94% (86; 100) respectively and was independent of the number of blocks scored (p = 0.315).

**Table 2:** Multivariable binary logistic regression models on discordance (yes/no) in histopathological growth pattern

Variable	Within metastasis (n = 702)		Between metastasis (n = 308)			
	OR (95% CI)	p-Value	Chemo-naive (n = 111)		Pre-treated (n = 197)	
			OR (95% CI)	p-Value	OR (95% CI)	p-Value
Location of primary						
Left versus right	1.513 (0.721–3.487)	0.298	0.610 (0.086–5.237)	0.620	0.655 (0.261–1.660)	0.367
Rectal versus right	1.881 (0.881–4.403)	0.120	3.174 (0.673–23.785)	0.186	0.604 (0.221–1.660)	0.324
pT3-4 versus pT0-2	1.259 (0.652–2.551)	0.506	1.163 (0.258–6.011)	0.848	1.193 (0.462–3.265)	0.721
Node-positive primary	0.650 (0.385–1.101)	0.107	0.516 (0.122–2.134)	0.354	0.696 (0.334–1.455)	0.332
Disease-free interval (months)^a^	0.998 (0.980–1.014)	0.852	0.995 (0.947–1.034)	0.828	1.017 (0.972–1.062)	0.434
Diameter of largest CRLM (cm)^a,b^	–	–	1.461 (1.073–2.145)	0.028*	1.139 (0.983–1.321)	0.081
Preoperative CEA (µg/L)^a^	0.999 (0.996–1.000)	0.152	0.996 (0.985–1.001)	0.278	1.000 (0.999–1.001)	0.575
Pre-treated versus chemonaive^c^	2.107 (1.231–3.679)	0.007*	–	–	–	–
Number of blocks scored						
3 versus 2	1.994 (1.140–3.441)	0.014*	–	–	–	–
4 versus 2	2.076 (0.788–4.869)	0.111	–	–	–	–
≥ 5 versus 2	1.418 (0.322–4.411)	0.589	–	–	–	–
Number of CRLM resected						
3 versus 2	–	–	0.868 (0.188–3.443)	0.846	3.602 (1.414–9.550)	0.008*
≥ 4 versus 2	–	–	1.617 (0.292–7.394)	0.548	5.887 (2.585–14.356)	< 0.001*

*OR* odds ratio, *CI* confidence interval, *CRLM* colorectal liver metastases, *CEA* carcinoembryonic antigen

^a^Continuous data entered into the model

^b^Omitted from within metastasis analysis since it is not representative for individual metastasis

^c^Used as stratification factor for between metastasis analysis

*α < 0.05

### Between metastasis concordance

Mean between metastasis concordance of all 363 patients was 90%. Since systemic treatment status was a significant predictor for within metastasis discordance, between metastasis analysis was performed in chemo-naive and pre-treated patients separately. Non-dHGP was found in 85% of chemo-naive patients versus 78% in pre-treated patients (p = 0.094). Results of the fitted multivariable logistic regression models on presence of HGP discordance (yes/no) amongst multiple resected CRLM are reported in Table 2. Within chemo-naive patients, the size of the largest hepatic tumour on preoperative imaging proved a significant predictor for between metastasis discordance with OR (95% CI) 1.461 (1.073; 2.145) and p = 0.028 for every cm increase in size. The only significant predictor found for between metastasis discordance in pre-treated patients was number of CRLM resected. Corresponding OR (95% CI) were 3.602 (1.414–9.550) for 3 versus 2 CRLM resected and 5.887 (2.585; 14.356) for ≥ 4 versus 2 CRLM resected (p = 0.008 and p < 0.001). Mean between metastasis concordance (Fig. 2b) was significantly lower in pre-treated versus chemo-naive patients (88% vs. 94%, p = 0.006). Figure 2b shows the mean between metastasis concordance for chemo-naive and pre-treated patients stratified by number of CRLM resected. In chemo-naive patients, mean between metastasis concordance [95% CI] did not differ amongst 2 (94% [91; 98]), 3 (94% [88; 99]) or ≥ 4 (90% [78; 100]) CRLM resected (p = 0.678). In pre-treated patients mean between metastasis concordance [95% CI] was significantly different amongst 2 (93% [90; 96]), 3 (85% [78; 92]) and ≥ 4 (83% [77; 88]) CRLM resected (p = 0.004).

### External validation

**Fig. 3:**
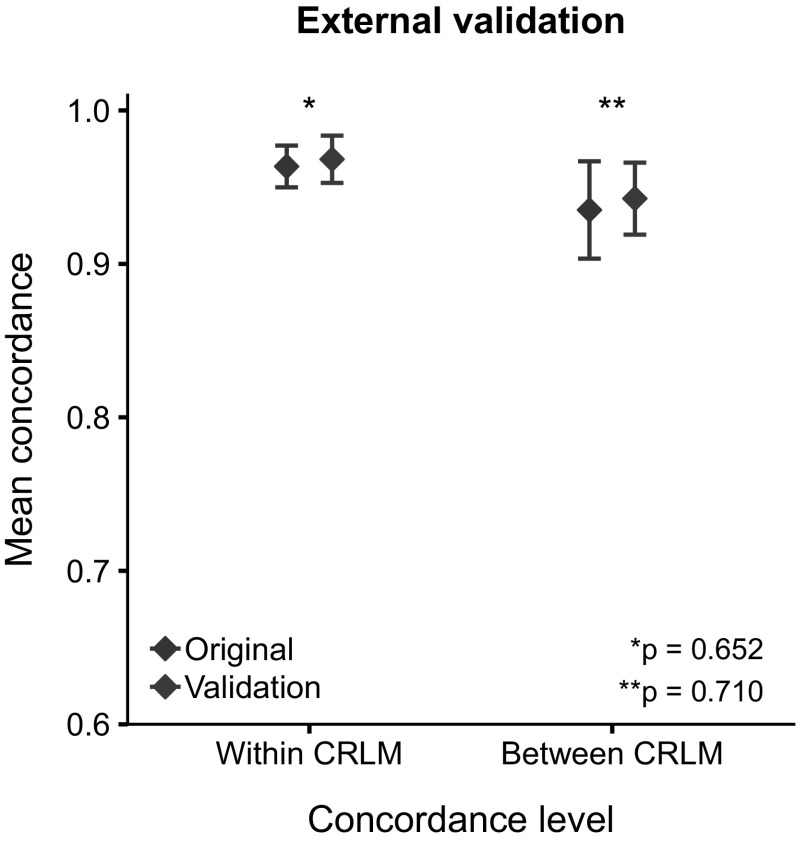
External validation of within and between colorectal liver metastasis (CRLM) concordance of histopathological growth pattern. Comparison was performed between the external validation cohort and (tissue of) chemonaive subjects from the original cohort

The external cohort comprised of 276 patients of whom the HGP could be determined in 251 (91%). In total 168 patients had resection performed of two or more CRLM and could be included for between metastasis analysis. Within metastasis analysis was performed in 270 CRLM with two or more blocks. Baseline characteristics were comparable between the external validation cohort and chemo-naive patients within the original cohort (Supplementary Table 1). Mean within (96% vs. 97%, p = 0.652) and between (94% vs. 94%, p = 0.710) metastasis concordance did not differ between the original (chemo-naive patients only) and validation cohort (Fig. 3).

### Diagnostic accuracy

Supplementary Fig. 1a displays the AUC for scoring a single (95.9%), two (97.7%) or three blocks (98.8%) per metastasis. A significant increase in diagnostic accuracy was observed for scoring 2 versus 1 block(s) (p = 0.039), but not for scoring 3 versus 2 blocks (p = 0.341). The AUC for scoring a single (93.3%), two (96.5%) or three (98.2%) resected CRLM per patient are reported in Supplementary Fig. 1b. A significant increase in diagnostic accuracy was found for scoring 2 versus 1 resected CRLM (p = 0.026), but not for scoring 3 versus 2 resected CRLM (p = 0.235).

### Learning curve

**Fig. 4:**
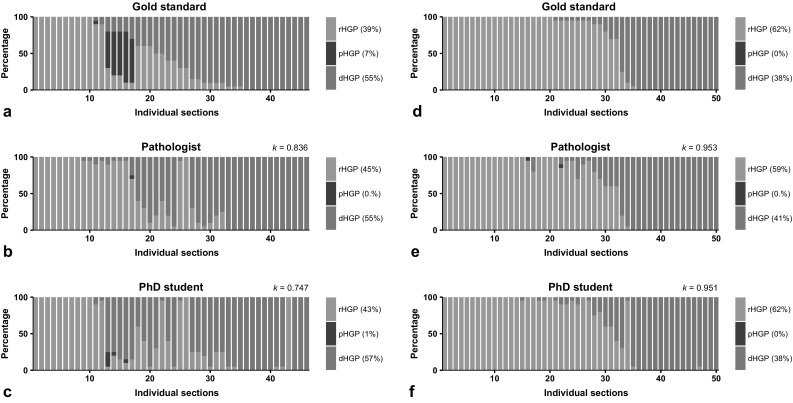
**a**, **d** Results of the first and second test-set as scored by the experienced trainer (gold standard). **b**, **e** Results of the first and second test-set as scored by the pathologist. **c**, **f** Results of the first and second test-set as scored by the PhD candidate. *rHGP* replacement type histopathological growth pattern (HGP), *pHGP* pushing type HGP, *dHGP* desmoplastic type HGP

The results of both test-sets as scored by the gold standard, the pathologist and the PhD candidate are graphically displayed in Fig. 4a–f. Interobserver agreement was higher in the second test-set for both the pathologist (*k *= 0.953 vs. *k *= 0.836) and the PhD candidate (*k *= 0.951 vs. *k *= 0.747). Where in the first test-set a difference in performance was seen between the pathologist and the PhD candidate (*k *= 0.836 and *k *= 0.747), performance in the second test-set did not differ (*k *= 0.953 and *k *= 0.951).

## Discussion

The current study found within metastasis concordance to be high (95%) when classifying the HGP as dHGP or non-dHGP. Furthermore, mean within metastasis concordance was independent of number of FFPE blocks scored. Overall between metastasis concordance was also high (90%), but differed for chemo-naive versus pre-treated patients (94% vs. 88%). In chemo-naive patients, mean between metastasis concordance was independent of number of CRLM resected and the only predictor found in multivariable analysis for discordance was size of largest hepatic tumour on preoperative imaging. For pre-treated patients, the number of CRLM resected proved predictive for between metastasis discordance. This finding was supported by a significant difference in mean concordance for 2, 3 or ≥ 4 resected CRLM within pre-treated patients.

External validation in a large cohort of chemo-naive patients found similarly high numbers of mean within (97%) and between (94%) metastasis concordance. Unfortunately, the external validation cohort comprised of chemo-naive patients only, as such external validation within pre-treated CRLM and patients could not be performed.

The current study suggests that systemic chemotherapy treatment prior to hepatic resection might somewhat affect the reliability of HGP assessment. In the same patient cohort as currently described, Galjart et al. reported a significant increase in dHGP within pre-treated patients [15]. It is as of yet unclear if this difference is due to chemotherapy directly changing HGP morphology, or due to selection bias in that patients with dHGP have improved prognosis and are thus more likely to complete their pre-operative chemotherapy and subsequent liver resection. Although inconclusive, the current study did find a higher heterogeneity amongst the HGP of slides and CRLM of pre-treated patients. This could be the result of chemotherapy having a direct effect on HGP morphology.

Two studies have previously reported on HGP concordance so far. Van Dam et al. analysed within metastasis agreement of ≥ 4 sections in a small sample of 50 CRLM [14] and Eefsen et al. reported on between metastases agreement in a small group of 24 patients with multiple resected CRLM [17]. As both studies applied different cut-off values to determine the HGP (50% and 75% respectively), interpretation of its results in light of the current study is difficult. Considering recent developments, it seems logical that future HGP classification will be based on the dHGP/non-dHGP cut-off.

When determining the diagnostic accuracy of HGP assessment, the current study found high AUC values for scoring a single, two or three blocks (all > 95%) or CRLM (all > 92%). The currently obtained results show that scoring two instead of one FFPE block(s) per CRLM increased diagnostic accuracy significantly. This increase was not significant when scoring three versus two blocks. As such, scoring two blocks per CRLM seems preferable and little accuracy is gained by further increasing the number of blocks assessed. This could significantly decrease workload, especially considering when non-dHGP is observed in a single block, the other available blocks of the same or different CRLM do not necessarily have to be assessed, for non-dHGP has readily been determined. Similar results were seen when looking at the diagnostic accuracy for scoring two versus one and three versus two CRLM resected in patients with multiple metastases. These findings suggest that CRLM treated by other modalities (e.g. ablative techniques) can accurately be diagnosed by CRLM resected within the same timeframe, especially in the case of two or more resected metastases.

Analysis of the learning curve showed that after a single training session by an experienced trainer good to excellent (*k *> 0.7) interobserver agreement for dHGP/non-dHGP was reached by two unexperienced observers. As expected, an observer with prior experience in liver pathology had a superior initial performance. After two training sessions however, the interobserver agreement was near perfect (*k *> 0.9) for both raters. Although only two unexperienced raters were included, these results suggest that HGP classification into dHGP or non-dHGP can be taught with relative ease and that interobserver agreement is high. In comparison, Chetty et al. reported on the interobserver agreement of tumour regression grade (TRG), a histopathological assessment within the field of colorectal cancer [27]. The overall agreement (expressed in *k*) was determined for three separate scoring systems: the Mandard [28], Dworak [29] and the modified rectal cancer regression grading system (m-RCRG) [30]. Seventeen experienced rectal cancer pathologists were asked to score ten slides of ten separate cases of rectal cancer treated with long-course preoperative chemoradiation. Reported overall agreement for the Mandard, Dworak and m-RCRG were *k *= 0.28, *k *= 0.35 and *k *= 0.38, respectively [27]. Furthermore, these results are also promising for automated HGP determination using digital image slides and ‘pathomics’, as it has shown great promise in other histological phenotypes [31]. Especially considering the new on/off phenomenon as described by Galjart et al. [15], automated determination on digital sections is something worth investigating and the authors feel could be feasible.

Common biomarkers used in clinical practice for the treatment of colorectal cancer include K-RAS and B-RAF mutational status. Richman et al. reported on within tumour heterogeneity of K-RAS and B-RAF in 69 primary CRC cases [32]. Intra-tumoural heterogeneity was found in 5/69 (7.2%) for K-RAS and 2/69 (2.9%) for B-RAF status [32]. When comparing multiple tumour sites, a recent meta-analysis by Bhullar et al. reported on the concordance of, amongst others, K-RAS and B-RAF between the primary tumour and its corresponding metastases [33]. Median biomarker concordance [range] for K-RAS and B-RAF were 93.7% [67–100] and 99.4% [80–100], respectively [33].

It appears that little within and between metastasis heterogeneity exists in the HGP of CRLM when classified as dHGP and non-dHGP. In addition, the observed heterogeneity seems comparable to that observed for biomarkers currently used in clinical practice. Furthermore, the diagnostic accuracy and learnability of HGP assessment by light microscopy seems high. These findings suggest that the HGPs of CRLM are a reliable and replicable histological biomarker.

## Electronic supplementary material

Below is the link to the electronic supplementary material.

Supplementary material 1 (PDF 254 kb)
