# Supplementary material for: Histopathological growth patterns of colorectal liver metastasis exhibit little heterogeneity and can be determined with a high diagnostic accuracy
Source: Clin Exp Metastasis. 2019 May 27;36(4):311–9. doi: 10.1007/s10585-019-09975-0 (PMC6611753; doi:10.1007/s10585-019-09975-0)
Supplement: Supplementary file 1 — Supplementary material 1 (PDF 254 kb) [file 10585_2019_9975_MOESM1_ESM.pdf]

Supplementary table 1. Comparison of baseline characteristics of chemo-naive patients in the original and external validation cohort included for between metastasis concordance analysis

		Cohort		
		Original	Validation	
		n=121 (%a)	n=168 (%a)	p-value
Gender	Female	75 (62)	107 (64)	0.717
	Male	46 (38)	60 (36)	
	Missing	0 (0)	1 (1)	
Age at resection CRLM - *(median [IQR])*		63.0 [60.0, 73.0]	63.0 [56.0, 69.0]	0.033
Primary tumour location	Colon	69 (58)	88 (53)	0.437
	Rectum	50 (42)	77 (47)	
	Missing	2 (2)	3 (2)	
T-stage	pT 0-2	29 (24)	26 (16)	0.072
	pT 3-4	91 (76)	140 (84)	
	Missing	1 (1)	2 (1)	
N-stage	N0	43 (36)	56 (34)	0.713
	N+	77 (64)	110 (66)	
	Missing	1 (1)	2 (1)	
Disease-free interval	>1 year	49 (40)	67 (40)	0.982
	≤1 year	72 (60)	99 (60)	
	Missing	0 (0)	2 (1)	
Diameter of largest CRLM	≤5 cm	105 (88)	84 (50)	<0.001
	>5 cm	15 (12)	83 (50)	
	Missing	1 (1)	1 (1)	
Preoperative CEA	≤200 µg/L	108 (92)	151 (90)	0.580
	>200 µg/L	9 (8)	16 (10)	
	Missing	4 (3)	1 (1)	
Histopathological growth pattern	dHGP	18 (15)	22 (13)	0.665
	non-dHGP	103 (85)	146 (87)	

CRLM: colorectal liver metastasis, IQR: interquartile range, CEA: carcinoembryonic antigen and (non-)dHGP: (non-)desmoplastic type histopathological growth pattern
a Percentages are expressed as proportions across each stratum (i.e. excluding missing). Percentages for missing are expressed as proportion of missing values within each stratum.

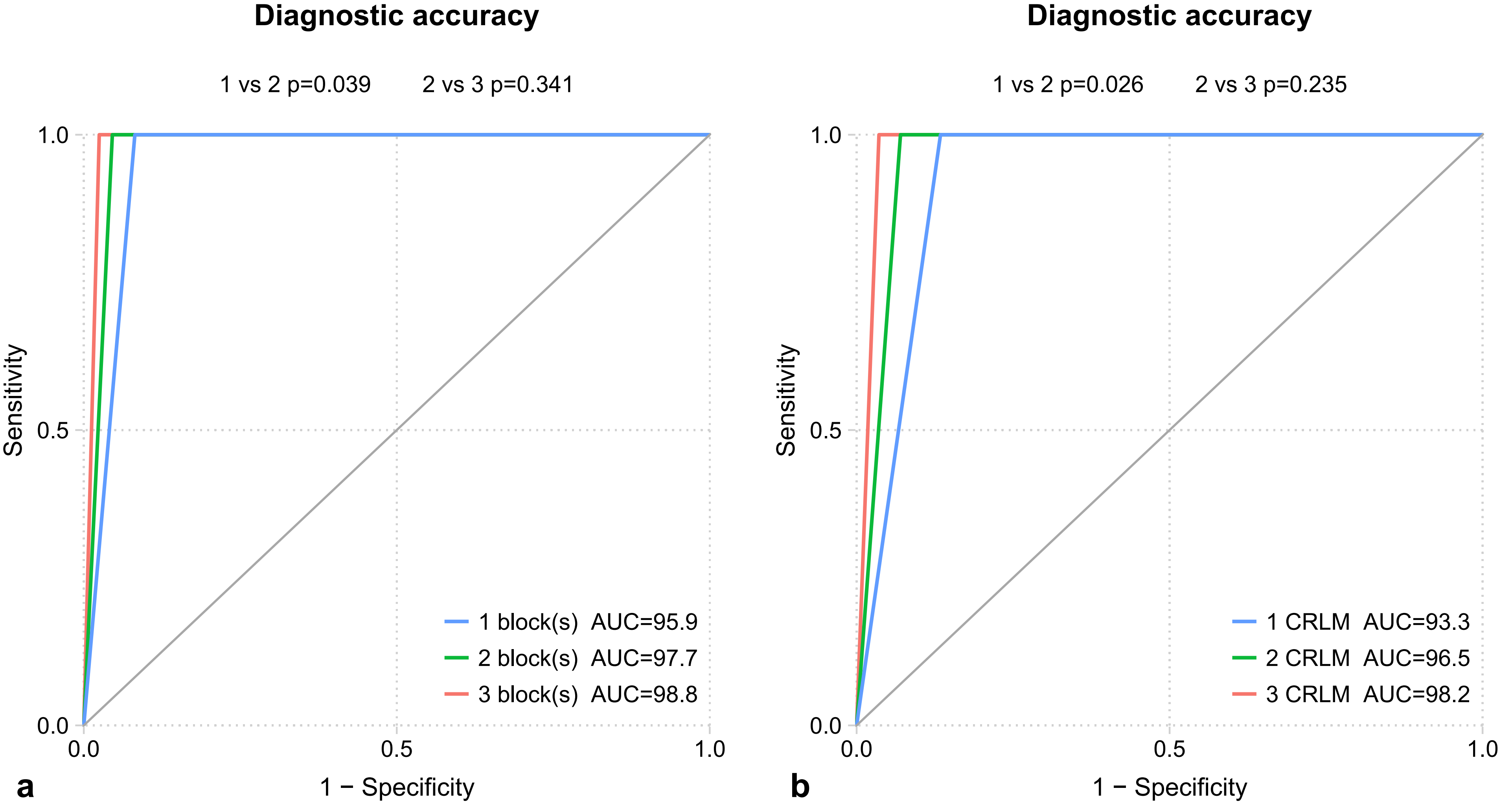

Supplementary figure 1: (a) Receiver operating characteristics (ROC) curves for determining metastasis histopathological growth pattern (HGP) by scoring 1, 2 or 3 block(s) respectively. (b) ROC curves for determining patient HGP by scoring 1, 2 or 3 resected CRLM respectively. *AUC: area under the curve.*
